# Advances in the Molecular Mechanisms of Cholangiocarcinoma: A Comprehensive Review of Biomarkers, Regulatory Pathways and Tumor Microenvironment Reprogramming

**DOI:** 10.3390/ijms27146362

**Published:** 2026-07-17

**Authors:** Yange Wang, Yanhua Yang, Meijing Wang, Zhonghua Liu, Meina Wang, Lu Zhang, Xiangqian Guo

**Affiliations:** 1 School of Basic Medical Sciences, Henan University, Kaifeng 475004, China; wangyange3@henu.edu.cn (Y.W.); liuzh@henu.edu.cn (Z.L.); 10190177@henu.edu.cn (M.W.); zhanglu9128@126.com (L.Z.); 2 Henan Provincial Engineering Center for Tumor Molecular Medicine, Kaifeng 475004, China; henuyang122@foxmail.com; 3 Henan Medical School, Henan University, Kaifeng 475004, China; 13994649458@163.com

**Keywords:** cholangiocarcinoma, biomarkers, molecular mechanisms, tumor microenvironment, precision medicine

## Abstract

Cholangiocarcinoma (CCA) is a malignant tumor arising from the epithelial cells of the biliary tract. It is characterized by high heterogeneity, frequent late diagnosis, high rates of metastasis, and overall poor prognosis. Unfortunately, the development of sensitive biomarkers and effective therapeutic approaches remains challenging and an unmet need. In recent years, advances in molecular biological techniques have significantly enhanced our understanding of the molecular mechanisms underlying CCA pathogenesis. This review summarized the biomarkers identified in CCA, highlighted the critical genes and signal transduction pathways closely associated with tumor cell proliferation and migration, inflammation regulation, angiogenesis, and tumor microenvironment, and discussed the complex regulatory roles of epigenetic alterations in CCA initiation and progression. These recent insights not only deepen our understanding of the molecular complexity of CCA but also lay a solid foundation for the development of precision medicine and individualized therapeutic strategies for patients with this aggressive malignancy.

## 1. Introduction

Cholangiocarcinoma (CCA), a malignant neoplasm arising from the epithelial cells of the biliary tract, has shown a rising global incidence, particularly in parts of Asia and certain Western countries [1]. Due to the lack of specific early symptoms, rapid disease progression, and limited therapeutic options, CCA usually has a dismal prognosis with a low five-year survival rate [2]. Therefore, a deeper understanding of the molecular mechanisms underlying CCA pathogenesis is essential for the development of effective preventive strategies, early diagnostic tools, and targeted therapies [3].

**Figure 1:**
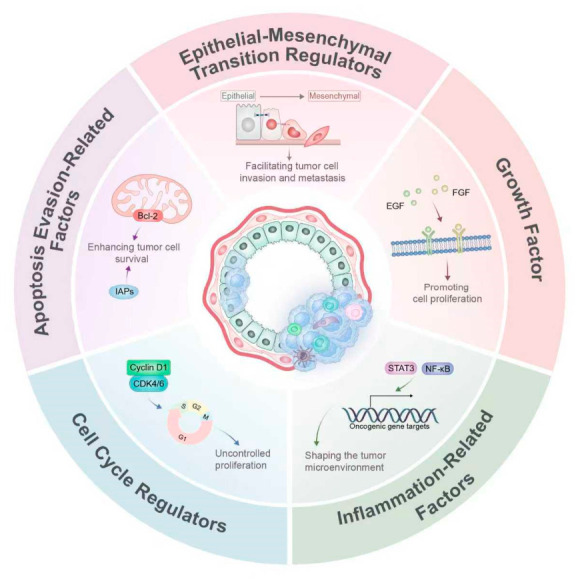
Mechanistic model of cholangiocarcinoma pathogenesis.

Over recent decades, the emergence of high-throughput technologies, such as gene microarrays, next-generation sequencing (NGS), and proteomics, has significantly advanced our understanding of the molecular basis of CCA progression [4]. These methods have enabled the identification of a number of genetic alterations and dysregulated molecules that play crucial roles in CCA initiation and development. Frequently reported molecules include growth factors (e.g., epidermal growth factor [EGF], fibroblast growth factor [FGF]) that promote cell proliferation; inflammation-related factors (e.g., NF-κB, STAT3) that shape the tumor microenvironment; cell cycle regulators (e.g., Cyclin D1, CDK4/6) that drive uncontrolled proliferation; anti-apoptotic factors (e.g., Bcl-2 family, IAPs) that enhance tumor cell survival; and epithelial–mesenchymal transition (EMT) regulators that facilitate tumor cell invasion and metastasis [3]. The intricate interplay among these molecules and their associated signaling pathways forms a highly interconnected network that collectively drives tumor cell proliferation, invasion, migration, and therapeutic resistance [4] (Figure 1).

Despite these advancements, several critical questions regarding the molecular signaling mechanisms in CCA remain unresolved. Do different subtypes of CCA (e.g., intrahepatic cholangiocarcinoma [iCCA], extrahepatic cholangiocarcinoma [eCCA], perihilar cholangiocarcinoma [pCCA] and distal cholangiocarcinoma [dCCA]) exhibit unique molecular signatures or activate distinct signaling pathways? How do environmental and genetic factors interact to modulate the activity of these signaling pathways? And, importantly, why do certain patients exhibit limited responses to current targeted therapies, and what are the underlying mechanisms of therapeutic resistance? Addressing these challenges requires deep, systematic and comprehensive investigation [4].

In light of these unanswered questions, this review aims to integrate current knowledge on the molecules and related signaling pathways involved in CCA progression, with a focus on their functional roles and regulatory mechanisms across different stages of this disease [1]. By critically evaluating the key literature and the latest research findings, we seek to elucidate the molecular basis of CCA pathogenesis and highlight promising therapeutic targets [5]. Additionally, we discuss the major challenges currently faced in this field and propose future research directions, aiming to provide a conceptual framework and practical guidance for advancing precision medicine and personalized treatment strategies in CCA [6].

Given the profound subtype-specific molecular heterogeneity across distinct anatomical forms of cholangiocarcinoma (CCA), this review primarily focuses on intrahepatic cholangiocarcinoma (iCCA) in accordance with current pathological classification consensus and accumulating multi-omics cohort evidence [7,8,9,10]. Although perihilar (pCCA) and distal (dCCA) cholangiocarcinoma are conventionally grouped together as extrahepatic CCA (eCCA) merely to facilitate clinical diagnosis and treatment management, robust genomic studies have confirmed that these two subtypes harbor distinct driver gene mutations, unique prognostic molecular signatures, and divergent tumor microenvironmental landscapes, rather than sharing homogeneous molecular features [11]. Distinctively, iCCA is enriched with therapeutically actionable molecular aberrations, most prominently *FGFR2* fusions and *IDH1/2* mutations. Further pathological subdivision of iCCA into small-duct and large-duct subtypes enables refined molecular stratification, laying a critical foundation for patient screening in targeted therapeutic interventions [12,13]. To date, multiple targeted drugs against *FGFR2* and *IDH1/2* alterations have received regulatory approval, and numerous ongoing clinical trials and translational studies have centered on iCCA, making its molecular landscape the most comprehensively characterized and clinically translatable among all CCA subtypes [9,10]. Accordingly, this review systematically dissects oncogenic signaling cascades, epigenetic remodeling, and stromal immune reprogramming mainly within the iCCA setting. Where comparative multi-omics and clinical data are available, we further elaborate on subtype-specific disparities in molecular alterations, aberrantly activated signaling axes, and tumor microenvironmental remodeling characteristics, as well as the corresponding differential therapeutic implications across iCCA, pCCA, and dCCA, so as to thoroughly address the molecular heterogeneity across anatomical CCA subtypes.

To achieve exhaustive and unbiased literature coverage for this review, we systematically queried PubMed, Web of Science, and Scopus for publications released through June 2026, using search terms encompassing cholangiocarcinoma, biomarkers, signaling pathways, tumor microenvironment, epigenetics, single-cell RNA sequencing, and exosomes. We prioritized peer-reviewed original research, systematic reviews, and authoritative consensus statements published between January 2015 and June 2026, with particular emphasis on recent mechanistic breakthroughs. Retrieved records were independently screened by title, abstract, and full text; case reports, conference abstracts, and cholangiocarcinoma studies lacking mechanistic insights were excluded. Additionally, we hand-searched the reference lists of key articles to identify any further eligible studies.

Several excellent reviews have comprehensively summarized specific aspects of cholangiocarcinoma biology, including the tumor immune microenvironment [14], cancer-associated fibroblast heterogeneity [15], epigenetic regulation [16], anatomic subtype classification [17], and, more recently, single-cell multi-omics approaches in biliary tract cancers and multi-omics biomarker discovery in intrahepatic cholangiocarcinoma [18,19]. However, most existing reviews focus on individual biological processes or specific technological platforms. In contrast, cholangiocarcinoma progression is increasingly recognized as a dynamic process driven by coordinated interactions among oncogenic signaling pathways, epigenetic remodeling, metabolic reprogramming, multicellular tumor microenvironment (TME) crosstalk, and spatial cellular heterogeneity. Therefore, rather than providing another descriptive summary of individual biomarkers, this review integrates these molecular mechanisms with emerging multi-omics technologies into a unified “mechanisms–microenvironment–technologies” framework, with a particular focus on intrahepatic cholangiocarcinoma. We believe this integrated perspective provides a more comprehensive understanding of disease progression while highlighting future opportunities for biomarker discovery and precision therapeutic strategies.

## 2. Key Biomarkers and Drivers of Cholangiocarcinoma Diagnosis, Prognosis and Progression

### 2.1. Biomarkers

Carbohydrate antigen 19-9 (CA19-9) is the most commonly used clinical biomarker for cholangiocarcinoma, with elevated serum levels observed in approximately 85% of patients with bile duct cancer [20]. However, its specificity is limited, as CA19-9 levels can also increase in pancreatic and gastric malignancies, as well as in severe hepatic injury [21]. Therefore, CA19-9 is primarily utilized for monitoring disease progression and evaluating therapeutic response in cholangiocarcinoma rather than for initial diagnosis [20].

In addition to CA19-9, other serum biomarkers such as carbohydrate antigen 125 (CA125) and carcinoembryonic antigen (CEA) are frequently elevated in CCA patients as well [22]. However, their diagnostic utility is similarly constrained by low specificity, as their levels may rise in intestinal inflammation, benign biliary obstruction, and gastrointestinal tumors, limiting their value in differentiating malignant from benign biliary lesions [23]. Alpha-fetoprotein (AFP), a classic biomarker for hepatocellular carcinoma, has limited but potential diagnostic relevance in cholangiocarcinoma [21], although its specificity and sensitivity in cholangiocarcinoma are inferior to those of CA19-9 [20].

In addition to the aforementioned conventional serum tumor biomarkers, a growing number of prognostic and treatment response biomarkers have been reported in cholangiocarcinoma in recent years. For instance, *TP53* is one of the most frequently mutated genes in cholangiocarcinoma and is closely linked to tumor initiation, progression, and patient prognosis [24]. Although less common, *KRAS* mutations are associated with an adverse prognosis of cholangiocarcinoma patients [20]. Overexpression of the anti-apoptotic protein Bcl-2 has been shown to inhibit cell apoptosis and is associated with a poor prognosis in cholangiocarcinoma [20]. Ki-67, a marker of cell proliferation, is valuable in differentiating benign from malignant intrahepatic biliary lesions, with high expression correlating with an adverse prognosis in intrahepatic cholangiocarcinoma [25]. Other reported prognostic biomarkers include cyclooxygenase-2 (COX-2), E-cadherin, SOX9, FZD10, VSNL1, PCP4, BUB1, and BUB1B [26]. Elevated COX-2 expression is associated with a poor prognosis in cholangiocarcinoma [27], whereas reduced expression of E-cadherin has been reported to be associated with tumor invasiveness and prognosis [28]. Moreover, epigenetic alterations such as hypermethylation of SOX9 and FZD10 have been identified as independent prognostic factors in cholangiocarcinoma [29].

### 2.2. Inflammatory Pathways

**Figure 2:**
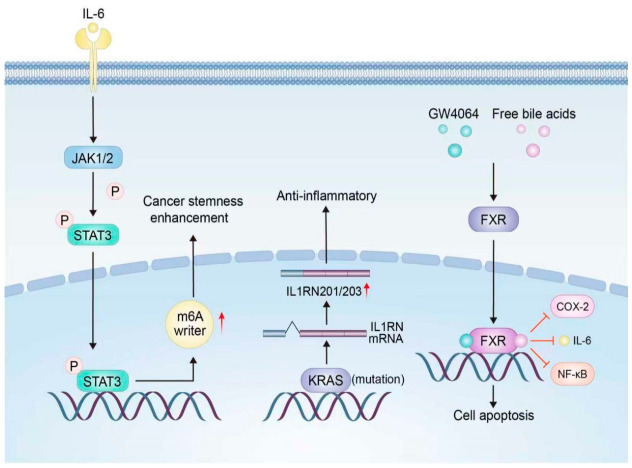
Schematic representation of inflammation-associated pathways in cholangiocarcinoma.

The tumor microenvironment plays pivotal roles in tumor initiation and progression. Cholangiocarcinoma cells and their surrounding stromal cells secrete various pro-inflammatory cytokines and growth factors, such as tumor necrosis factor (TNF)-α, interleukin (IL)-6, transforming growth factor (TGF)-β, EGF, and platelet-derived growth factor (PDGF), which have been reported to drive tumor growth, invasion, and angiogenesis through autocrine and paracrine signaling mechanisms [30] (Figure 2).

IL-6 is a key pro-inflammatory cytokine that promotes cholangiocyte proliferation and activates the p44/p42 MAPK signaling pathway [31]. Furthermore, the binding of IL-6 to its receptor complex (IL-6R/gp130) activates the JAK/STAT3 signaling cascade, inducing the activation of JAK1/2 and subsequent phosphorylation and activation of STAT3 [32]. STAT3, a key transcription factor in the IL-6 signaling cascade, plays a crucial role in the development and progression of cholangiocarcinoma [33]. Notably, interstitial cells in the tumor microenvironment have been reported to secrete inflammatory factors such as IL-6, IL-1, and TNF-α, which activate STAT3 and its downstream genes in tumor epithelial cells, thereby promoting tumor progression [34]. COX-2, an enzyme upregulated by inflammatory factors such as TNF-α and IL-6, contributes to early tumor formation [3]. As previously discussed, high COX-2 expression is indicative of a poor prognosis in cholangiocarcinoma, highlighting its roles in inflammation regulation and tumor progression [3].

Furthermore, emerging evidence implicates RNA modifications, particularly N6-methyladenosine (m6A), as playing crucial roles in the initiation and progression of cholangiocarcinoma. The IL-6/STAT3 axis has been shown to regulate m6A writer genes, which enhance cancer stemness in cholangiocarcinoma [35]. By direct binding to the promoter regions of m6A writer genes, STAT3 can induce m6A writer gene expression in cholangiocarcinoma [35]. This discovery highlights the importance of m6A modification in inflammatory regulation and the maintenance of tumor stemness in cholangiocarcinoma [35].

Recent research by Fan Jia and Gao Qiang from Fudan University has elucidated a critical link between oncogenic *KRAS* mutations and pro-tumorigenic inflammation in iCCA. Their work reveals a dual-phase mechanism: while mutant *KRAS* drives a potent pro-inflammatory response, it concurrently triggers a compensatory negative feedback loop. Specifically, the *KRAS*-mutated iCCA cells upregulate specific splicing isoforms of the interleukin-1 receptor antagonist (primarily IL1RN-201/203) through alternative splicing. These isoforms are secreted and function as soluble decoys, antagonizing the IL-1 signaling pathway and thereby exerting significant anti-inflammatory effects. Therapeutically, this self-regulating loop represents a druggable inflammatory checkpoint. Enhancing the IL1RN pathway with exogenous IL-1RA (Anakinra) can potentiate this natural feedback, reprogram the immunosuppressive tumor microenvironment, and synergize with anti-PD-1 immunotherapy. These findings delineate a sophisticated interplay between an oncogenic driver and inflammatory regulation, revealing a novel therapeutic vulnerability in *KRAS*-mutated iCCA [36].

Bile acids, traditionally recognized for their roles as metabolic regulators in cholesterol metabolism and lipid digestion, also function as signaling molecules that regulate tumor progression. Unconjugated bile acids upregulated the farnesoid X receptor (FXR), whereas conjugated bile acids downregulated it [37]. Co-treatment of cholangiocarcinoma cells with unconjugated bile acids and the FXR agonist GW4064 resulted in significant inhibition of cell proliferation [38], accompanied by suppressed activity of IL-6, COX-2, and NF-κB, ultimately leading to blocked tumor growth-promoting signaling pathways and induced cell apoptosis [39]. These findings suggest that targeting FXR signaling may represent a promising therapeutic strategy for cholangiocarcinoma.

In addition to the pathways and molecules described above, several other inflammatory molecules are aberrantly expressed in CCA and contribute to the development and progression of cholangiocarcinoma. For example, elevated expression of COX-2, *EGFR*, HBx protein, CK19, and PCNA is closely related to inflammatory responses and tumor development in cholangiocarcinoma [40], and is often associated with malignancy, poor prognosis, and treatment resistance in cholangiocarcinoma [3].

### 2.3. Cell Proliferation and Metastasis Pathways

**Figure 3:**
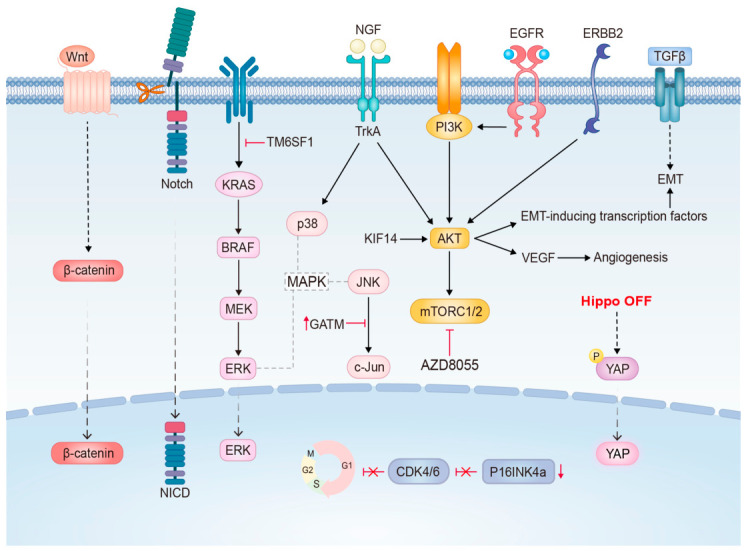
Schematic representation of critical genes and pathways involved in cholangiocarcinoma proliferation and metastasis. This diagram depicts nine major interconnected signaling cascades frequently dysregulated in CCA tumorigenesis: the Wnt/β-catenin, Notch, Ras–MAPK, PI3K/Akt/mTOR, JNK, Hippo/YAP, NGF/TrkA, *EGFR*/ERBB2, and TGF-β pathways. Essential regulatory molecules alongside the mTOR inhibitor AZD8055 are highlighted to delineate their biological roles in cell cycle progression, malignant proliferation, angiogenesis, and epithelial–mesenchymal transition (EMT). Solid arrows indicate direct molecular regulatory interactions; dashed arrows represent indirect regulatory events; and gradient-colored dashed arrows illustrate the cytoplasm-to-nucleus translocation of functional effector proteins. Cytoplasmic YAP is marked with a phosphorylation tag to indicate its transcriptionally inactive state. Upon Hippo pathway inactivation (labeled Hippo OFF), dephosphorylated YAP is released from cytoplasmic retention and translocates into the nucleus to drive the transcription of oncogenic target genes. Persistent aberrant activation of these intertwined signaling networks collectively facilitates the proliferation, invasiveness, and metastatic dissemination of CCA cells.

Cholangiocarcinoma is characterized by the aberrant activation of multiple signaling pathways that collectively drive tumor cell proliferation, cell cycle progression, apoptosis, EMT, invasion, and metastasis. The key oncogenic cascades, including the Ras-MAPK, PI3K-AKT-mTOR, JNK, Notch, Wnt/β-catenin, Hippo/YAP, NGF/TrkA, *FGFR*4, and TGF-β pathways, have been reported to drive oncogenic processes either alone or synergistically by promoting cell cycle progression, inhibiting apoptosis, enhancing survival signaling, and facilitating EMT and invasiveness. Central regulatory molecules, including *KRAS*, *TP53*, KIF14, MCM2, p16INK4a, DPC4, Ki-67, TM6SF1, DEK, *EGFR*, and ERBB2, function as oncogenes or tumor suppressors to modulate the above pathways (Figure 3). Dysregulation of these molecular cascades not only contributes to malignant transformation but also influences clinical prognosis and therapeutic response, highlighting their potential as targets for precision medicine in cholangiocarcinoma.

The Ras-MAPK signaling pathway is a central driver in the pathogenesis and progression of cholangiocarcinoma. This pathway is frequently activated by mutations in key upstream molecules such as *KRAS* and BRAF [41]. Aberrant activation promotes uncontrolled cell proliferation and survival and often cross-talks with other pro-oncogenic cascades, particularly the PI3K-AKT-mTOR axis, to fuel tumor growth and progression [42].

*KRAS* mutations, one of the most common genetic alterations in CCA, serve as a critical oncogenic driver, leading to constitutive pathway activation and promoting tumor cell proliferation and malignant progression [43]. In contrast, TM6SF1 has been identified as a putative tumor suppressor that inhibits the *KRAS*-MEK-ERK pathway. Its downregulation enhances proliferation, migration, and invasion while suppressing apoptosis in intrahepatic cholangiocarcinoma, correlating with poor patient prognosis [44]. Additionally, KIF14 contributes to tumor proliferation through functional interaction with AKT signaling, underscoring its role as a key effector within this regulatory network of proliferation [45,46].

*TP53* is a critical tumor suppressor gene frequently inactivated in cholangiocarcinoma [47]. Mutations in *TP53* impair DNA damage repair function, resulting in unchecked cell cycle progression, enhanced cell proliferation, and eventual malignant transformation [47]. Abnormal p53 protein expression is observed in approximately 52.8% of cholangiocarcinoma cases, and overexpression of p53 is inversely correlated with survival rates in cholangiocarcinoma patients with primary sclerosing cholangitis (PSC) [48]. Additionally, MCM2, a downstream molecule of p53, contributes to CCA tumor progression by accelerating cell cycle progression and inhibiting apoptosis.

The cyclin-dependent kinase inhibitor p16INK4a functions as a negative regulator of the G1/S checkpoint by inhibiting CDK4/6 kinases, thereby preventing the cell cycle transition from G1 to S phase, and leading to suppressed cell proliferation [49]. Inactivation of p16INK4a is frequently observed in cholangiocarcinoma and is often accompanied by nuclear accumulation of β-catenin, implicating it in both cell cycle regulation and canonical Wnt pathway activation during perihilar cholangiocarcinoma development [50]. Similarly, defects in the DPC4 gene have been reported to accelerate the progression from G1 to S phase, thereby enhancing cell proliferation capacity in CCA [50]. Ki-67, a well-established marker of cell proliferation, is highly expressed in intrahepatic cholangiocarcinoma tissues, serving as a valuable diagnostic and prognostic tool for distinguishing benign from malignant lesions in perihilar cholangiocarcinoma [25].

The JNK pathway plays crucial regulatory roles in the progression of cholangiocarcinoma by regulating cell proliferation, apoptosis, and malignancy. As an oncogenic signaling axis, its activation promotes tumor growth and aggressive behavior. The study by Yu et al. further highlights that the novel biomarker GATM exerts its tumor-suppressive effects specifically by attenuating this pathway. GATM overexpression was shown to inhibit the JNK/c-Jun signaling axis, leading to suppressed proliferation and reduced malignancy of cholangiocarcinoma cells, thereby positioning the JNK pathway as a critical central node for both pro-tumorigenic signals and therapeutic interventions [51].

The Notch pathway plays critical roles in regulating the differentiation, repair, and growth of biliary cells. In cholangiocarcinoma, the expression of receptors such as Notch1, Notch3, and Notch4 is often upregulated, with Notch3 expression correlating with disease severity [52]. The crosstalk between the Notch pathway and the PI3K/AKT pathway can further amplify proliferation signals to create a synergistic oncogenic loop [52]. However, therapeutic targeting of Notch must be approached cautiously, as its function exhibits a stark tissue-specific contrast: it promotes progression in cholangiocarcinoma, whereas it suppresses growth in hepatocellular carcinoma (HCC) and predicts a better prognosis in the latter [53].

The canonical Wnt/β-catenin pathway regulates hepatic and biliary development and cell proliferation. In cholangiocarcinoma, activation of the Wnt/β-catenin pathway plays critical roles in the initiation, progression and development of drug resistance in CCA [54]. Notably, non-canonical Wnt signaling can also stimulate cholangiocyte proliferation independent of β-catenin [55]. Emerging Wnt inhibitors have shown promising anti-tumor efficacy in cholangiocarcinoma mouse models and are currently under evaluation in clinical trials, implicating the therapeutic potential of targeting the Wnt signaling pathway [55].

The Hippo/YAP signaling pathway is a critical regulator of cell proliferation and tissue homeostasis. However, in cholangiocarcinoma, its effector YAP is frequently activated through Hippo-independent signals such as inflammatory cytokines (e.g., IL-6) and growth factors (e.g., PDGF, FGF). Activated YAP then translocates to the nucleus to promote the transcription of pro-proliferation and pro-invasion genes. Given that the Hippo pathway integrates inputs from multiple upstream signals rather than a single dedicated receptor, it serves as a central signaling hub, underscoring its attractiveness as a therapeutic target for combination treatments [56]. Beyond its canonical role as the terminal effector of the Hippo pathway, YAP is increasingly recognized as a central signaling hub that integrates multiple intrinsic and extrinsic oncogenic cues in cholangiocarcinoma. YAP activity is shaped by crosstalk with receptor tyrosine kinase signaling, Wnt-associated pathways, inflammatory mediators, and microenvironmental signals, thereby coordinating tumor proliferation, survival, and therapeutic resistance [57,58,59,60,61]. Consistent with this concept, YAP/TAZ serve as key sensors of mechanotransduction and inflammatory signaling, linking diverse microenvironmental inputs to oncogenic pathway activation [61]. Although this convergence makes YAP an attractive therapeutic target, compensatory activation of parallel signaling networks may limit the efficacy of YAP-directed monotherapies. These observations support the development of combination strategies targeting YAP together with its interconnected signaling pathways to achieve more durable therapeutic responses [57,58,59,60,61].

The PI3K/AKT/mTOR signaling pathway is one of the most frequently dysregulated pathways in human cancers, including cholangiocarcinoma, and it regulates critical processes such as cell proliferation, apoptosis, autophagy, and metastasis [62]. AZD8055, a dual mTOR inhibitor targeting mTORC1/2, has been shown to effectively suppress the proliferation and migration of cholangiocarcinoma cells [63] by inhibiting mTOR kinase activity and consequently blocking the phosphorylation of its downstream effectors, including AKT (at Ser473), S6, and 4EBP1 [64]. The dual kinase inhibition of mTORC1/2 impairs both survival and proliferation (via AKT and MAPK suppression), as well as migration processes (by decreased expression of MMP2 and MMP9), exhibiting superior anti-tumor capacity compared with selective single-target inhibitors in cholangiocarcinoma [63].

The activation of the NGF/TrkA signaling pathway engages the MAPK/Erk/P38 and PI3K/AKT cascades, thereby enhancing the proliferation and invasiveness of intrahepatic cholangiocarcinoma cells [65]. Additionally, *FGFR*4 activation significantly enhances the proliferation, migration, and EMT of cholangiocarcinoma cells [66].

The TGF-β signaling pathway plays dual roles in cancer: it acts as a tumor suppressor in early stages but promotes invasion, metastasis, and immune evasion in advanced disease. In cholangiocarcinoma, TGF-β signaling drives EMT and associated fibrogenic responses to promote tumor invasion and metastasis, making it a potential therapeutic target for intervention strategies [67].

Members of the receptor tyrosine kinase family (RTKs), particularly *EGFR* (epidermal growth factor receptor) and ERBB2 (also known as HER2), are frequently overexpressed in cholangiocarcinoma and are associated with advanced stages, high histological grade, metastasis, and poor prognosis [68]. Overexpression of these receptors leads to the constitutive activation of downstream signaling pathways that promote tumor growth, vascularization, and metastasis [68]. Thus, inhibition of c-Cbl-mediated degradation of *EGFR* sustains receptor accumulation and maintains its activation, leading to increased expression of downstream VEGF and EMT-inducing transcription factors, thereby promoting angiogenesis and the EMT program [68]. Therefore, targeting *EGFR* and ERBB2 represents a promising therapeutic strategy for cholangiocarcinoma treatment [68].

Collectively, these findings support a network-based understanding of *KRAS*/MAPK signaling in cholangiocarcinoma: rather than functioning as an isolated oncogenic pathway, MAPK signaling appears to operate within an interconnected regulatory ecosystem shaped by reciprocal interactions between tumor cells and the TME. Single-cell and spatial transcriptomic studies have revealed that tumor cells, tumor-associated macrophages (TAMs), cancer-associated fibroblasts (CAFs), endothelial cells, and other stromal populations form spatially organized tumor niches that promote disease progression and therapeutic resistance [69,70,71]. The recently identified ZDHHC5–BRAF–ERK axis further exemplifies how post-translational regulation can coordinate MAPK signaling with other oncogenic programs through shared molecular nodes [72]. This network-based conceptualization suggests that targeting MAPK signaling alone may be insufficient, as compensatory immune and stromal interactions within the TME may sustain tumor progression. Therefore, combining MAPK inhibitors with immune- or stromal-targeted therapies may offer a more effective therapeutic strategy than single-agent MAPK inhibition [69,70,71,72,73].

### 2.4. Angiogenesis and Tumor Microenvironment in CCA

**Figure 4:**
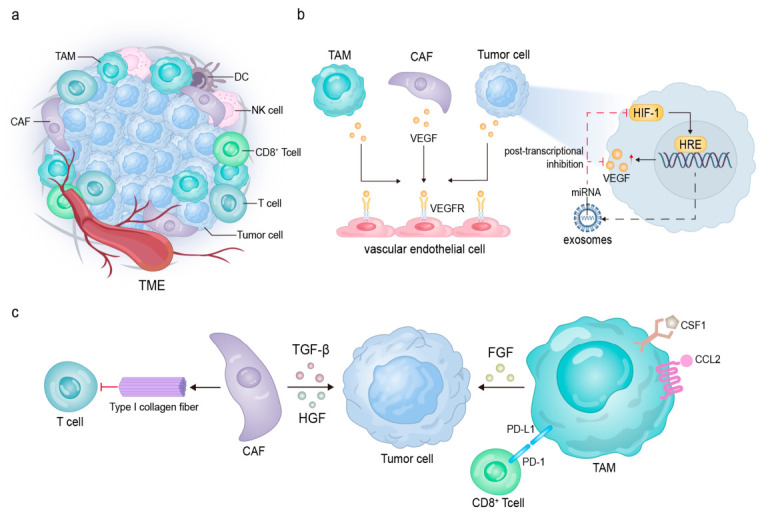
Schematic illustration of angiogenesis pathways and the tumor microenvironment in cholangiocarcinoma. (**a**) Cellular landscape of the CCA TME, consisting of tumor cells, cancer-associated fibroblasts (CAFs), tumor-associated macrophages (TAMs), dendritic cells (DCs), natural killer (NK) cells, and multiple subtypes of T lymphocytes. (**b**) Mechanism underlying VEGF-dependent tumor angiogenesis. CAFs, TAMs and malignant tumor cells act as the predominant cellular sources of VEGF, which engages vascular endothelial growth factor receptor (VEGFR) on vascular endothelial cells to trigger pathological angiogenesis. Under hypoxic conditions, hypoxia-inducible factor-1 (HIF-1) binds to hypoxia-response elements (HREs) to transcriptionally upregulate *VEGF* expression in tumor cells. Tumor cells also release microRNAs (miRNAs) encapsulated within exosomes; the dashed arrows denote that exosomal miRNAs indirectly repress the HIF-1/VEGF signaling axis at the post-transcriptional level. (**c**) Immunosuppressive regulatory networks within the CCA TME. CAFs secrete transforming growth factor-β (TGF-β) and hepatocyte growth factor (HGF) to remodel the extracellular matrix and accelerate tumor malignant progression. Tumor-derived CSF1 and CCL2 drive the recruitment and phenotypic polarization of TAMs. These reprogrammed TAMs further mediate CD8^+^ T cell exhaustion and establish an immune-suppressive tumor milieu via activating the PD-L1/PD-1 immune checkpoint pathway.

The tumor microenvironment (TME) refers to the local milieu in which tumor cells reside, encompassing not only the tumor cells themselves but also a complex network of surrounding stromal cells, extracellular matrix (ECM), blood vessels, immune cells, and various signaling molecules. As a dynamic and evolving system, the TME undergoes continuous remodeling during tumor progression [74]. It plays a crucial role not only in tumor growth and metastasis but also in therapeutic response and clinical outcomes [75] (Figure 4).

The tumor microenvironment plays pivotal roles in the initiation and progression of tumors. The vasculature in the TME is essential for sustaining the metabolic demands of rapidly growing tumors, and is critical for cell proliferation and tumor growth. In intrahepatic cholangiocarcinoma, high expression of vascular endothelial growth factor (VEGF) indicates a poor prognosis [76], and this high level of VEGF promotes cell proliferation, survival, and migration, driving the development of a pro-angiogenic microenvironment [76].

Both VEGF and its receptor VEGFR are key regulators of both physiological and pathological angiogenesis. Activation of this axis enhances vascular permeability, recruits endothelial progenitor cells, and facilitates tumor perfusion, thereby supporting tumor expansion and dissemination and strongly influencing patient prognosis [77]. Hypoxia, a hallmark of solid tumors, induces the stabilization, accumulation, and dimerization of Hypoxia-inducible factor-1 (HIF-1) to functionally bind to hypoxia response elements (HREs) in the VEGF promoter, thereby further activating VEGF gene expression and angiogenesis [78]. In addition to VEGF, HIF-1 regulates the expression of other angiogenic factors, including PDGF-β, Ang-1, and Ang-2 [78]. Notably, Ang-2 and VEGF can promote the migration and invasion of spheroids derived from hepatocellular carcinoma and intrahepatic cholangiocarcinoma, processes closely linked to EMT, a key mechanism of tumor metastasis. Additionally, MACC1 has been reported to upregulate VEGFA to promote angiogenesis and tumor progression in cholangiocarcinoma [79,80]. TROP2 can also promote cell proliferation, clonal formation, and tumor angiogenesis [81]. Under hypoxic conditions, cholangiocarcinoma cells can exploit intercellular communication via exosomes to shape the vascular niche. Specifically, tumor-derived exosomes can transfer their cargo (such as proteins, nucleic acids, lipids, and metabolites) to endothelial cells, impairing endothelial barrier function and enhancing angiogenic capacity by targeting key regulatory genes. These findings highlight the importance of angiogenesis in cholangiocarcinoma, offering potential therapeutic targets within the vascular microenvironment for this malignancy [82] (Figure 4).

Cancer-associated fibroblasts are among the most abundant stromal cell types in CCA, typically expressing α-smooth muscle actin (α-SMA) [83]. CAFs contribute to tumor progression through multiple mechanisms: they secrete hepatocyte growth factor (HGF), which activates the c-MET receptor in tumor cells to promote tumor cell proliferation and migration/invasion; produce excessive type I collagen fibers to form a dense fibrotic stroma that acts as a fibrotic barrier to restrict T-cell infiltration and exert immunosuppressive effects [84]; and release pro-angiogenic and growth factors such as VEGF and TGF-β to promote tumor cell proliferation, invasion and tumor angiogenesis [85]. CAFs have been reported to be deeply involved in tumor growth, progression, and chemoresistance [86]. Clinically, high α-SMA expression in the tumor stroma is correlated with reduced survival rates in ICC patients [87].

Tumor-associated macrophages are another critical component of the CCA microenvironment, predominantly exhibiting an M2-like (pro-tumor) phenotype. TAMs are recruited to the TME via chemokines such as CSF1 and CCL2 and are activated by signals from tumor and stromal cells. Once residing within the TME, TAMs promote tumor progression by secreting cytokines such as CXCL14, IL-8, IL-13, VEGF, and FGF, which promote angiogenesis, recruit inflammatory cells, and promote tumor cell survival [88].

Importantly, TAMs play central roles in immune suppression within the TME. They contribute to T cell exhaustion and the establishment of immune tolerance by expressing, or being induced to express, immune checkpoint ligands such as PD-L1, which engages PD-1 on cytotoxic CD8^+^ T cells, leading to their functional impairment [89]. Additionally, TAMs promote the expansion of CD4^+^CD25^+^FOXP3^+^ T regulatory cells, further dampening anti-tumor immunity [89]. The FOXP3^+^ Treg cell population represents a major barrier to effective antitumor immunity, and recent advances have highlighted FOXP3 as a central transcription factor governing Treg identity and function, with emerging therapeutic strategies targeting Treg depletion or FOXP3 modulation being explored in various malignancies [90].

The interplay between tumor cells, CAFs, TAMs and immune checkpoints defines the immunosuppressive landscape of CCA and contributes to its resistance to conventional therapies and immunotherapy. Targeting these interactions paves promising avenues for the development of targeted therapies for cholangiocarcinoma [89]. To provide a structured overview of the key biomarkers and signaling pathways discussed above, we summarize their biological roles, evidence levels, subtype distributions, and clinical relevance in Table 1 at the end of this paper.

## 3. Epigenetic Regulation

Epigenetic mechanisms, encompassing DNA methylation, histone modifications, non-coding RNAs, and chromatin remodeling, play central roles in fundamental biological and pathological processes, including embryonic development, cell fate determination, and tumorigenesis. Aberrant epigenetic modifications can lead to the activation of oncogenes or suppression of tumor suppressor genes, thereby driving tumor initiation and progression [114]. In particular, non-coding RNAs exert extensive regulatory functions on gene expression through both transcriptional and post-transcriptional epigenetic mechanisms, offering new insights into the molecular pathogenesis of CCA and identifying potential biomarkers and therapeutic targets [115].

### 3.1. DNA Methylation Associated with Cholangiocarcinoma Development

DNA methylation, particularly CpG island methylation, typically represses gene transcription. Thus, aberrant promoter hypermethylation of tumor suppressor genes or hypomethylation of oncogenes are frequently observed in cholangiocarcinoma, regulating malignant transformation and often leading to poor clinical outcomes.

Several genes have been identified as DNA methylation targets with diagnostic or prognostic significance in CCA. FZD10, which encodes a cell surface receptor for Wnt molecules, is another methylation-driven gene that regulates tumor occurrence and correlates with cholangiocarcinoma prognosis [29]. NPTX2 promoter methylation is significantly more prevalent in bile samples from cholangiocarcinoma patients than in those from patients with common bile duct stones. Furthermore, the diagnostic potential of methylation markers in liquid biopsies has been demonstrated. For example, a dual-marker panel assessing NPTX2 and FOXE1 promoter methylation in bile has been shown to significantly improve the sensitivity and specificity for the early detection of cholangiocarcinoma compared with single-marker assays [91].

Genome-wide methylation profiling using the 850K methylation array has revealed a total of 12,259 differentially methylated CpG sites in CCA, of which 78% were hypermethylated. This hypermethylation landscape suggests a global repressive chromatin state in CCA. Among the aforementioned candidates, DNA methylation of cg27362525 and cg26597242 and the expression of genomic concomitant genes including DEPDC1, FUT4, MDK, PACS1, GCNT1, PIWIL4, miR-22, and miR-551b could be further explored as potential biomarkers for cholangiocarcinoma [91]. GCNT1 has been identified as a methylation-driven gene in CCA, with its expression being negatively regulated by the methylation status of a specific CpG site (cg27362525). Its elevated expression in tumor tissue and association with patient prognosis support its potential as a prognostic biomarker [91].

### 3.2. Histone Modifications Associated with Cholangiocarcinoma Development

Histone modifications, including methylation, acetylation, phosphorylation, and ubiquitination, dynamically regulate chromatin structure, gene accessibility, and expression. In intrahepatic cholangiocarcinoma, active histone marks such as H3K4me3, H3K4me1, and H3K27ac are frequently altered in the enhancer and promoter regions of critical genes, contributing to dysregulated transcription programs [16]. The transcription factor AP-1, composed of Jun and Fos proteins, forms homo- or heterodimers that bind specific DNA sequences to regulate target gene expression [116]. Importantly, AP-1 acts as a key epigenetic mediator by recruiting histone-modifying enzymes to specific genomic loci, thereby facilitating an open chromatin state and promoting the transcription of genes involved in cell proliferation, differentiation, and inflammation—processes central to cholangiocarcinoma development [16]. P300 is a histone acetyltransferase that catalyzes the acetylation of the H3K27 site, thereby promoting an open chromatin conformation and facilitating gene transcription [16]. In CCA, P300 binds to the promoter region of the *METTL16*, a methyltransferase involved in RNA metabolism, and catalyzes H3K27 acetylation at this locus, further leading to enhanced *METTL16* transcription and increased expression [92]. *METTL16* expression is positively correlated with the level of H3K27 acetylation at the *METTL16* gene. Elevated *METTL16* expression, in turn, promotes cholangiocarcinoma cell proliferation and tumor progression. This P300-H3K27ac-METTL16 axis highlights a critical epigenetic regulatory circuit in the development of cholangiocarcinoma and suggests that targeting P300 or its downstream effectors may represent a viable therapeutic strategy for this malignancy [92].

### 3.3. Non-Coding RNAs Relevant to the Development of Cholangiocarcinoma

Non-coding RNAs, particularly miRNAs and long non-coding RNAs (lncRNAs), are increasingly recognized as key regulators of CCA biology, influencing tumor initiation, survival, progression, metastasis, and drug resistance through diverse epigenetic and post-transcriptional mechanisms. For instance, miR-21 is upregulated in CCA tissues and cell lines, can function to promote tumor survival and proliferation, and its inhibition induces apoptosis in CCA cells, suggesting its oncogenic and potential therapeutic roles [93]. Acting as a downstream mediator of the tumor-associated lncRNA FALEC, miR-20a-5p directly targets SHOC2 to inhibit the proliferation, migration, and invasion of CCA cells in vitro, and also mediates the ERK1/2 signaling pathway, contributing to 5-FU resistance [117]. In contrast, upregulation of miR-199a-3p can enhance the cisplatin sensitivity in cholangiocarcinoma cells by inhibiting the expression of the multidrug resistance gene 1 (MDR1) [94].

LncRNAs play significant and diverse regulatory roles in cholangiocarcinoma progression through various molecular mechanisms. The lncRNA HULC functions as a competitive endogenous RNA (ceRNA) that sequesters miRNAs, leading to the upregulation of C-X-C chemokine receptor 4 (CXCR4), thereby promoting CCA cell migration and invasion [95]. LncRNA ASAP1-IT1 has been reported to promote the proliferation and migration of cholangiocarcinoma cells, although its precise molecular mechanism requires further elucidation [118]. In contrast, the lncRNA MEG3 acts as a tumor suppressor by increasing doxorubicin sensitivity through the downregulation of drug efflux transporters ABCG2 and MRP1, thereby enhancing intracellular drug accumulation [96]. LncRNA TP73-AS1 is overexpressed in CCA and associated with poor clinical outcomes; it exhibits oncogenic properties by promoting cell proliferation and inhibiting apoptosis both in vitro and in vivo [119]. LncRNA PVT1 promotes proliferation and migration by epigenetically repressing ANGPTL4 expression, potentially through the recruitment of histone-modifying complexes [97]. LncRNA ATB promotes growth and metastasis by acting as a ceRNA for miR-200c, a known suppressor of EMT and metastasis, thereby enhancing tumor aggressiveness [120]. Additionally, LINC01061 functions as a molecular sponge for miR-612, leading to the upregulation of SEMA4D and subsequent promotion of tumor growth in CCA [120].

### 3.4. Chromatin Remodeling in Cholangiocarcinoma Pathogenesis

Chromatin remodeling constitutes a core epigenetic machinery governing chromatin accessibility, transcriptional programming, and genomic integrity in an ATP-dependent manner. This process is mediated by multi-subunit complexes falling into four primary families: SWI/SNF, ISWI, CHD, and INO80/SWR, whose dysregulation fuels the initiation and advancement of cholangiocarcinoma (CCA) [121]. Among these families, the SWI/SNF complex remains the most extensively characterized, and its functional impairment is tightly implicated in CCA tumorigenesis. Under physiological circumstances, intact SWI/SNF assemblies sustain regular nucleosome positioning and grant transcriptional machinery access to target genomic loci, thereby maintaining biliary epithelial homeostasis and restraining malignant transformation. Conversely, loss-of-function mutations disrupting SWI/SNF subunits trigger oncogenic transformation of biliary epithelial cells [98].

Recurrent inactivating variants in *PBRM1*—the gene encoding the SWI/SNF subunit BAF180—are frequently identified in intrahepatic cholangiocarcinoma (iCCA) [98]. Depletion of *PBRM1* destabilizes intact SWI/SNF complexes and abolishes their intrinsic tumor-suppressive capacity [98]. Mechanistically, *PBRM1* deficiency instigates functional antagonism between SWI/SNF and PRC2 complexes. This interaction elevates genome-wide H3K27me3 deposition at the promoters of tumor-suppressive genes, silencing downstream anti-tumor transcriptional programs [99]. This molecular crosstalk has laid the groundwork for a clinically validated synthetic lethal therapeutic strategy: pharmacological inhibition of EZH2 via tazemetostat yields sustained therapeutic responses and extends overall survival among patients with metastatic *PBRM1*-mutant CCA, underscoring the translational value of the SWI/SNF–PRC2 regulatory axis [99]. Beyond *PBRM1*, mutations targeting other SWI/SNF subunits also contribute to CCA progression. *SMARCA4* (BRG1) encodes the ATPase catalytic core of SWI/SNF; its somatic aberrations are prevalent across multiple human malignancies, and loss of *SMARCA4* expression correlates with unfavorable clinical outcomes in CCA patients [100]. Protein arginine methyltransferase 5 (PRMT5) further intersects with chromatin remodeling networks by modulating a broad spectrum of chromatin regulators and DNA repair mediators. Pharmacological suppression of PRMT5 impedes the proliferation of patient-derived CCA organoids, concurrently triggering R-loop accumulation and DNA double-strand breaks, which directly links perturbed chromatin remodeling to genomic instability [101]. Multi-omics profiling has further demonstrated that dysfunctional chromatin remodelers drive aberrant enhancer activation, wherein dysregulated chromatin accessibility enables unrestrained transcription of oncogenic drivers [122].

Collectively, these cumulative findings establish chromatin remodeling as the fourth central epigenetic regulatory layer, alongside DNA methylation, histone post-translational modifications, and non-coding RNA signaling. Disordered chromatin architecture, epigenetic landscape reprogramming, accumulated genomic instability, and hyperactivated oncogenic enhancers jointly mediate CCA tumorigenesis driven by remodeler dysfunction [123]. Key epigenetic mediators involved in these cascades, including EZH2 and PRMT5, represent promising candidate biomarkers and actionable therapeutic targets. Future investigations are warranted to dissect subtype-specific epigenetic vulnerabilities to refine targeted epigenetic regimens for CCA treatment.

The epigenetic regulatory machinery, including DNA methylation, histone post-translational modifications, non-coding RNAs, and chromatin remodeling, engages in reciprocal crosstalk with core oncogenic signaling cascades to drive cholangiocarcinoma (CCA) progression and the emergence of therapeutic resistance. At the transcriptional level, DNA methylation and histone modifications reprogram the activity of signaling pathways, with the Wnt/β-catenin cascade serving as a representative example: epigenetic upregulation of pathway repressors including *AXIN1* and *GSK3B* dampens oncogenic signal transduction and mitigates chemotherapy resistance [124]. Chromatin remodelers further tune signaling outputs by governing the accessibility of transcription factors to pathway-associated genes. For instance, loss-of-function mutations in *PBRM1* compromise the structural integrity of SWI/SNF complexes, abolishing their inhibitory crosstalk with PRC2. This event triggers excessive genome-wide H3K27me3 accumulation, rewires global chromatin accessibility, and rewrites the transcriptional landscape of downstream target genes [125]. At the post-transcriptional level, non-coding RNAs precisely calibrate signaling responses via direct targeting of core effector molecules linked to gemcitabine and cisplatin insensitivity. The miR-182-5p/ADK/SEMA5a regulatory axis exemplifies this mode of regulation [126], whereas miR-27a-3p targets the FoxO signaling pathway to confer malignant phenotypes on biliary epithelial cells [127]. Taken together, these cumulative observations indicate that combinatorial inhibition of epigenetic modulators and oncogenic signaling hubs represents a highly viable therapeutic strategy to reverse drug resistance in CCA.

Collectively, epigenetic dysregulation through the aforementioned mechanisms—DNA methylation, histone modification, chromatin remodeling, and non-coding RNAs—plays a central role in the pathogenesis of cholangiocarcinoma. These mechanisms not only drive tumor progression but also contribute to diagnostic challenges and therapeutic resistance. Importantly, many of these epigenetic alterations are reversible and detectable in liquid biopsies, making them promising candidates for early detection, prognostic stratification, and targeted therapy. Future research should focus on translating these findings into clinically applicable epigenetic biomarkers and therapies.

## 4. Advanced Technologies Applied in Cholangiocarcinoma Research

Recent advances in multi-omics and single-cell technologies have revolutionized our understanding of cholangiocarcinoma biology, enabling high-resolution dissection of tumor heterogeneity, microenvironmental complexity, and non-invasive biomarker discovery. Single-cell transcriptomics, spatial transcriptomics, radiogenomics, and exosome-based liquid biopsy are transforming CCA from a histologically defined malignancy into a molecularly stratified disease with actionable therapeutic targets.

### 4.1. Single-Cell RNA Sequencing

Recent advances in single-cell RNA sequencing (scRNA-seq) have revolutionized our understanding of CCA intratumoral heterogeneity and have opened opportunities to identify rare cell populations and develop potential targeted therapies in CCA. Through scRNA-seq, distinct cell subtypes of CCA have been identified, displaying cell type-prominent processes including chromatin remodeling, metabolism, and chronic inflammation, each associated with unique biological behaviors and relative clinical outcomes. For instance, APOE^+^C1QB^+^ TAMs are associated with poor prognosis in CCA [102] due to their immunosuppressive properties and resistance to immunotherapy, highlighting their potential as therapeutic targets [102]. Another breakthrough is the discovery of MAL2, a tetraspan-like transmembrane protein overexpressed in CCA cells, which promotes lipid metabolism reprogramming via the *EGFR*/PI3K/AKT/SREBP-1 axis and contributes to chemoresistance [103]. These discoveries were enabled by scRNA-seq. Inhibition of MAL2 enhances cisplatin sensitivity, supporting its role as both a biomarker and a therapeutic vulnerability [103]. Additionally, a subpopulation of Tm4sf1^high^ malignant cells has been identified as cancer stem-like cells (CSCs) with dynamic interactions in the tumor microenvironment, inducing immune evasion and tumor progression [128]. Immunogenic cell death (ICD)-related signatures derived from scRNA-seq have identified an ICD-high subtype of CCA with increased immune infiltration but paradoxically worse outcomes, mediated by the ANXA-FPR axis that drives macrophage polarization [129]. Furthermore, SPP1 (osteopontin), identified as a prognostic biomarker in CCA, interacts with CD4^+^ T cells to suppress antitumor immunity via TGF-β signaling, thereby facilitating immune escape [104,105]. APOC1 has been identified as a regulator of G0/G1 cell cycle arrest in CCA, linking quiescent cancer cell populations to therapeutic resistance and recurrence [130]. These scRNA-seq-based findings underscore the power of this technology in uncovering novel biomarkers that refine CCA classification and guide precision therapies.

### 4.2. Spatial Transcriptomics

While scRNA-seq analyzes the cell composition of bulk tumor tissues, spatial transcriptomics (ST) adds a critical geographic dimension to the analysis of cell crosstalk in the tumor microenvironment. ST has identified distinct tumor–stroma–immune triads in the invasive margin of iCCA, where POSTN^+^FAP^+^ CAFs, SPP1^+^ macrophages, and CD8^+^ T cells form an immunosuppressive niche that promotes tumor invasion [71]. Notably, mucosal-associated invariant T (MAIT) cells recruit SPP1+ macrophages via chemokine signaling, while endothelial cells enhance stromal crosstalk, creating a physical barrier that limits immune infiltration [71]. ST-based classification stratified iCCA into five spatial subtypes with distinct prognoses, among which CD163^hi^ M2-like macrophages are key mediators of immune suppression through direct interaction with exhausted CD8+ T cells [131]. Additionally, TFF3, secreted by malignant subclones, reprograms macrophages toward a protumorigenic polarization, further amplifying immune evasion [70]. These insights were uncovered by spatial transcriptomics. Spatial mapping also revealed CXCL5 and SLC6A14 as biomarkers of microvascular invasion, and serum CXCL5 levels are associated with metastasis risk [106]. Furthermore, CTSE^+^ tumor cells co-localized with MARCO^+^ macrophages, forming an immune-resistant niche enriched in LGALS9–CD44 signaling, which is associated with intrahepatic metastasis and poor survival [132]. Similarly, DAB2^+^ macrophages and FAP^+^ CAFs collaborate to establish an immune-excluded barrier in both iCCA and HCC, highlighting conserved stromal–immune crosstalk across iCCA and liver cancers [133]. These findings underscore ST’s power in uncovering spatially informed biomarkers and therapeutic targets, refining patient stratification in CCA.

### 4.3. Radiogenomics

Mounting evidence demonstrates that the integration of CT-based radiomic features with genomic data can effectively predict molecular characteristics and clinical outcomes in CCA, offering novel non-invasive solutions for this highly heterogeneous malignancy.

A landmark study by Ji et al. identified a robust three-gene signature (*PLAUR*, *CD40LG*, and *FGFR4*) through the integration of spatial transcriptomics and radiomics data. This signature demonstrated prognostic potential and remarkable accuracy (AUC = 0.84) in predicting response to immunochemotherapy, validated in a multicenter cohort of 331 patients. Notably, spatial mapping revealed the predominant localization of these biomarkers within the tumor epithelium rather than the stroma. In particular, preclinical studies have shown that patients with high PLAUR expression may benefit from combined uPAR inhibitor and PD-1 blockade therapy [107], providing critical insights for targeted therapeutic strategies [107]. The radiomic models developed by Viganò et al. [108] achieved impressive accuracy in predicting molecular alterations, with AUC values of 0.89 for *FGFR2* fusions and 0.82 for *IDH1* mutations, significantly outperforming conventional clinical parameters and enabling non-invasive genotyping. Huang and Chen [134] further elucidated the correlation between imaging phenotypes and molecular subtypes in iCCA, identifying distinct radiologic patterns associated with inflammation-rich, proliferative, and metabolic molecular subgroups. Despite the remaining challenges in standardizing feature extraction protocols and establishing multicenter validation frameworks [135], radiogenomics represents a paradigm shift in CCA management, elevating diagnostic imaging from a descriptive tool to a predictive, quantitative discipline that bridges the gap between non-invasive diagnosis and targeted therapeutic intervention.

### 4.4. Exosome

Exosomes, nanoscale extracellular vesicles ranging from 30 to 150 nm in diameter, are secreted by tumor and stromal cells and carry molecular cargo (proteins, nucleic acids) that can reflect the state of their cell of origin [136]. In CCA, exosome-based liquid biopsies offer a non-invasive means of early detection, prognosis prediction, and real-time monitoring of therapeutic response. Beyond exosomes, the broader family of extracellular vesicles (EVs) plays multifaceted roles in remodeling the tumor immune microenvironment through intercellular transfer of proteins, lipids, and nucleic acids, offering additional avenues for biomarker discovery and therapeutic intervention [137]. Recent studies have highlighted the translational potential of exosome-based biomarkers in CCA diagnosis, prognosis, and therapeutic monitoring, and exosomal circular RNAs (circRNAs), miRNAs, lncRNAs, and proteins have demonstrated superior diagnostic performance compared with conventional serum biomarkers such as CA19-9. For instance, Wen et al. identified exosomal circRNA signatures in bile and serum with diagnostic AUROCs of 0.947 (bile) and 0.861 (serum), respectively, and established a circRNA-based prognostic model for early recurrence prediction in CCA patients undergoing curative-intent surgery (bile-ERS C-index = 0.783; serum-ERS C-index = 0.782) [109]. Similarly, bile exosomal miR-21-5p demonstrated superior diagnostic performance for biliary tract cancer detection, with an AUC of 0.913, significantly outperforming both serum miR-21-5p (AUC = 0.628) and the conventional serum biomarker CA19-9 (AUC = 0.793) [110]. Exosomal Cripto-1 levels have also been shown to correlate with metastasis and poor survival in perihilar cholangiocarcinoma, further supporting the prognostic utility of exosomal biomarkers in CCA [111]. Mechanistically, exosomes mediate tumor progression through intercellular communication, circulating tumor cell-derived exosomal TTN-AS1 promotes CCA cell proliferation and migration [138], and exosomal miR-3124-5p enhances angiogenesis by targeting GDF11 [139]. Although challenges remain in isolation standardization, cargo quantification, and clinical validation, exosome-based liquid biopsies represent a paradigm shift toward non-invasive, real-time monitoring of CCA, offering unprecedented opportunities for precision oncology.

## 5. Conclusions and Perspective

Cholangiocarcinoma is driven by a complex interplay of genetic alterations and dysregulated molecular signaling pathways. This review has summarized recent advances in understanding the molecular basis of CCA pathogenesis, highlighting potential therapeutic targets for CCA intervention.

The emerging evidence collectively supports a paradigm shift in understanding CCA pathogenesis: rather than operating as linear, independent cascades, multiple oncogenic pathways are organized into an interconnected signaling network characterized by functional redundancy, cross-activation, and convergence on shared downstream effectors [112,113]. This network architecture is exemplified by the observation that, despite substantial genetic heterogeneity, intrahepatic CCA growth remains dependent on a limited repertoire of pathways converging on common nodes such as ERK and PI3K [112]. Organoid-based models have further demonstrated that growth factor signaling and MAPK pathway activation are coordinately active in CCA, with kinase inhibitor screening revealing “pan-effective” inhibitors that reflect functional redundancy and compensatory activation within the network [113]. The PI3K/AKT/mTOR axis is highly regulated through multiple cross-interactions with diverse signaling cascades, underscoring its role as a central network hub rather than an isolated effector [140]. Critically, recent therapeutic advances targeting molecular subsets such as FGFR2 fusions and *IDH1* mutations have underscored that understanding pathway interactions—rather than merely identifying individual oncogenic drivers—is essential for overcoming treatment bottlenecks [141]. These findings carry profound therapeutic implications: isolated targeting of a single pathway may be inherently limited by network redundancy and compensatory cross-activation, whereas rationally designed combination regimens that simultaneously intercept multiple network nodes hold greater promise for achieving durable clinical responses. Most of these findings align with the classical Hallmarks of Cancer framework, such as sustaining proliferative signaling, resisting cell death, and inducing angiogenesis, underscoring their consistent relevance in CCA biology. However, it is noteworthy that emerging hallmarks proposed by Hanahan [142], including polymorphic microbiomes and the role of senescent cells, have been less extensively explored compared with other hallmarks in CCA.

This relative paucity may reflect the distinct biological and etiological characteristics of CCA. First, the mutational landscape of CCA (e.g., high-frequency mutations in *IDH1*, IDH2, *KRAS*, and *FGFR*2) directly activates the classical oncogenic pathways, potentially diminishing the relative contribution of broad non-genetic modulation to tumor initiation and progression. Second, the investigation into emerging dimensions like the intratumoral and gut microbiomes remains at an early stage, with limited evidence on their functional impact and clinical relevance in CCA; thus, more attention and mechanistic studies are necessary. These observations reinforce the universality of the Hallmarks of Cancer as a conceptual framework, while also highlighting the tumor- and tissue-specific nuances in how these hallmarks are manifested; therefore, a deeper understanding of such context-dependent tumor biology is critical for the development of tailored therapeutic strategies for CCA.

While significant progress has been made, substantial gaps remain in understanding molecular heterogeneity, targeted therapies, and treatment resistance. Future research is needed to elucidate inter- and intra-patient molecular heterogeneity and to clarify how oncogenic molecular variations, environmental exposures, and metabolic reprogramming converge to shape CCA development and progression.

Clinically, the limited efficacy of current targeted therapies underscores the urgent need for innovative treatment strategies. Rational combinations of targeted agents, immunotherapies, and stromal-modulating drugs hold promise for overcoming therapeutic resistance. Moreover, targeting epigenetic processes, such as inhibitors of methylation, histone modification, and chromatin remodeling, represents a particularly promising frontier for both diagnostic and therapeutic innovation.

In conclusion, advancing CCA research requires multidisciplinary integration of genomics, immunology, microbiology, and systems biology. By elucidating the molecular architecture of CCA and its dynamic interaction with the TME, we can accelerate the translation of mechanistic insights into effective, personalized therapies and ultimately improve the outcomes for patients suffering from this aggressive and therapeutically challenging disease.

## Figures and Tables

**Table 1:** Key biomarkers and pathways in cholangiocarcinoma: clinical utility, biological roles, and subtype distribution.

Biomarker/Pathway	Biological Role	Evidence Type	CCA Subtype	Clinical Relevance	Ref.
CA19-9	Sialylated glycan; tumor-associated antigen	Retrospective cohort	All	Diagnostic and Prognostic	[20,21]
CA125/CEA	Glycoproteins; tumor-associated antigens	Retrospective cohort	All	Diagnostic	[22,23]
AFP	Alpha-fetoprotein; classic HCC biomarker	Retrospective cohort	All	Diagnostic	[20,21]
*TP53*	Tumor suppressor; regulates cell cycle and apoptosis	Retrospective cohort	All	Prognostic	[24]
*KRAS*	Oncogenic GTPase; drives MAPK signaling	Retrospective cohort	iCCA (*KRAS*-mutated)	Prognostic	[20,36]
Bcl-2	Anti-apoptotic protein; inhibits apoptosis	Retrospective cohort	Not specified	Prognostic	[20]
Ki-67	Cell proliferation marker	Retrospective cohort	iCCA, pCCA	Diagnostic and Prognostic	[25]
COX-2	Enzyme; inflammation regulation	Retrospective cohort	Not specified	Prognostic	[3,27]
E-cadherin	Cell adhesion molecule; EMT regulator	Retrospective cohort	Not specified	Prognostic	[28]
SOX9/FZD10	Transcription factor/Wnt receptor; epigenetic targets	Retrospective cohort (methylation analysis)	Not specified	Prognostic	[29]
IL-6/STAT3	Pro-inflammatory cytokine/transcription factor	Preclinical	Not specified	Therapeutic	[31,32,33,34,35]
m6A writers	RNA modification enzymes; regulate cancer stemness	Preclinical	Not specified	Therapeutic	[35]
GATM	Novel biomarker; tumor suppressor	Preclinical	Not specified	Therapeutic	[51]
Notch pathway	Regulates differentiation, repair, and growth	Preclinical	Not specified	Therapeutic	[52,53]
Wnt/β-catenin pathway	Regulates hepatic/biliary development and proliferation	Preclinical; clinical trials (Wnt inhibitors)	Not specified	Therapeutic	[54,55]
Hippo/YAP pathway	Central signaling hub; integrates multiple oncogenic cues	Preclinical	Not specified	Therapeutic	[56,57,58,59,60,61]
PI3K/AKT/mTOR pathway	Regulates proliferation, apoptosis, autophagy, metastasis	Preclinical	Not specified	Therapeutic	[62,63,64]
NGF/TrkA pathway	Activates MAPK/Erk/P38 and PI3K/AKT cascades	Preclinical	iCCA	Therapeutic	[65]
FGFR4	Receptor tyrosine kinase	Preclinical	Not specified	Therapeutic	[66]
TGF-β pathway	Dual role: tumor suppressor (early) vs. pro-metastatic (advanced)	Preclinical	Not specified	Therapeutic	[67]
EGFR/ERBB2 (HER2)	Receptor tyrosine kinases	Retrospective cohort	Not specified	Prognostic and Therapeutic	[68]
VEGF/VEGFR	Key regulators of angiogenesis	Retrospective cohort	iCCA	Prognostic and Therapeutic	[76,77,78]
MACC1	Regulates VEGFA upregulation	Preclinical	Not specified	Therapeutic	[79,80]
TROP2	Cell surface glycoprotein	Preclinical	Not specified	Therapeutic	[81]
Exosomal miRNAs	Intercellular communication; regulate angiogenesis	Preclinical	Not specified	Diagnostic and Therapeutic	[82]
CAFs (α-SMA+)	Stromal cells; secrete HGF, VEGF, TGF-β	Retrospective cohort	iCCA	Prognostic and Therapeutic	[83,84,85,86,87]
TAMs (M2-like)	Immune cells; secrete CXCL14, IL-8, IL-13, VEGF, FGF	Retrospective cohort; scRNA-seq	Not specified	Prognostic and Therapeutic	[88,89,90]
FZD10 methylation	Wnt receptor; methylation-driven gene	Methylation array analysis	Not specified	Prognostic	[29]
NPTX2/FOXE1 methylation	Dual methylation markers in bile	Prospective cohort (liquid biopsy)	Not specified	Diagnostic	[91]
P300-H3K27ac-METTL16 axis	Epigenetic regulatory circuit; histone acetylation	Preclinical	Not specified	Therapeutic	[92]
miR-21	Oncogenic miRNA; promotes survival/proliferation	Preclinical	Not specified	Therapeutic	[93]
miR-199a-3p	Tumor suppressor miRNA	Preclinical	Not specified	Therapeutic	[94]
lncRNA HULC	ceRNA; sequesters miRNAs	Preclinical	Not specified	Therapeutic	[95]
lncRNA MEG3	Tumor suppressor lncRNA	Preclinical	Not specified	Therapeutic	[96]
lncRNA PVT1	Oncogenic lncRNA; epigenetic repressor	Preclinical	Not specified	Therapeutic	[97]
*PBRM1* (SWI/SNF)	Chromatin remodeler; tumor suppressor	Retrospective cohort; clinical case report	iCCA	Prognostic and Therapeutic	[98,99]
*SMARCA4* (BRG1)	ATPase catalytic core of SWI/SNF	Retrospective cohort	Not specified	Prognostic	[100]
PRMT5	Protein arginine methyltransferase	Preclinical (patient-derived organoids)	Not specified	Therapeutic	[101]
APOE^+^C1QB^+^ TAMs	Immunosuppressive macrophage subtype	scRNA-seq	Not specified	Prognostic and Therapeutic	[102]
MAL2	Tetra-transmembrane protein; lipid metabolism reprogramming	scRNA-seq	Not specified	Prognostic and Therapeutic	[103]
SPP1 (Osteopontin)	Immunosuppressive molecule	scRNA-seq	Not specified	Prognostic and Therapeutic	[104,105]
POSTN^+^FAP^+^ CAFs/SPP1^+^ macrophages/CD8^+^ T cells	Immunosuppressive niche at invasive margin	Spatial transcriptomics	iCCA	Prognostic and Therapeutic	[71]
TFF3	Secreted by malignant subclones; reprograms macrophages	Spatial transcriptomics	Not specified	Therapeutic	[70]
CXCL5/SLC6A14	Biomarkers of microvascular invasion	Spatial transcriptomics	iCCA	Prognostic	[106]
PLAUR/CD40LG/FGFR4	3-gene radiogenomic signature	Radiogenomics (multicenter cohort, AUC = 0.84)	iCCA	Predictive	[107]
*FGFR2* fusions/*IDH1* mutations	Actionable molecular aberrations	Radiogenomics (AUC = 0.89 for FGFR2; AUC = 0.82 for *IDH1*)	iCCA	Predictive and Therapeutic	[9,10,108]
Exosomal circRNAs	Diagnostic and recurrence monitoring	Prospective cohort (liquid biopsy)	Not specified	Diagnostic and Prognostic	[109]
Bile-derived exosomal ncRNAs	Non-invasive biomarkers	Prospective cohort	Not specified	Diagnostic and Prognostic	[110]
Exosomal Cripto-1	Potential biomarker	Preclinical	pCCA	Diagnostic	[111]
Organoid models	Patient-derived 3D cultures; drug screening	Preclinical (organoid-based)	Not specified	Predictive	[112,113]

Definition of Evidence Level. Clinical: Evidence derived from human serum, bile or surgical tumor specimens, pathological protein staining and gene sequencing data, validated via clinical cohorts and registered clinical trials. Preclinical: In vitro cell experiments, organoid culture and xenograft animal model data.

## Data Availability

No new data were created or analyzed in this study. Data sharing is not applicable to this article.

## Abbreviations

The following abbreviations are used in this manuscript:

AFP	Alpha-fetoprotein
α-SMA	alpha-smooth muscle actin
CA125	Carbohydrate antigen 125
CA19-9	Carbohydrate antigen 19-9
CAF	Cancer-associated fibroblast
CCA	Cholangiocarcinoma
CEA	Carcinoembryonic antigen
ceRNA	Competitive endogenous RNA
circRNA	Circular RNA
COX-2	Cyclooxygenase-2
CSC	Cancer stem-like cell
CXCR4	C-X-C chemokine receptor 4
dCCA	Distal cholangiocarcinoma
eCCA	Extrahepatic cholangiocarcinoma
ECM	Extracellular matrix
EGF	Epidermal growth factor
EGFR	Epidermal growth factor receptor
EMT	Epithelial–mesenchymal transition
FGF	Fibroblast growth factor
FXR	Farnesoid X receptor
HCC	Hepatocellular carcinoma
HGF	Hepatocyte growth factor
HIF-1	Hypoxia-inducible factor-1
HRE	Hypoxia response element
iCCA	Intrahepatic cholangiocarcinoma
ICD	Immunogenic cell death
IHC	Immunohistochemistry
lncRNA	Long non-coding RNA
m6A	N6-methyladenosine
MAIT	Mucosal-associated invariant T
MDR1	Multidrug resistance gene 1
NGS	Next-generation sequencing
pCCA	Perihilar cholangiocarcinoma
PDGF	Platelet-derived growth factor
PSC	Primary sclerosing cholangitis
RTK	Receptor tyrosine kinase family
scRNA-seq	Single-cell RNA sequencing
ST	Spatial transcriptomics
TAM	Tumor-associated macrophage
TGF	Transforming growth factor
TME	Tumor microenvironment
TNF	Tumor necrosis factor
VEGF	Vascular endothelial growth factor
VEGFRs	VEGF receptors
